# Prevalence and the associated factors of hepatitis B and hepatitis C viral infections among HIV-positive individuals in same-day antiretroviral therapy initiation program in Bangkok, Thailand

**DOI:** 10.1186/s12889-021-12429-6

**Published:** 2022-01-20

**Authors:** Supanat Thitipatarakorn, Tanat Chinbunchorn, Jitsupa Peelay, Pich Seekaew, Sorawit Amatavete, Monsiri Sangsai, Tippawan Pankam, Anchalee Avihingsanon, Matthew Avery, Praphan Phanuphak, Reshmie Ramautarsing, Nittaya Phanuphak

**Affiliations:** 1 Institute of HIV Research and Innovation, 319 Chamchuri Square Building, Fl. 11, Unit 1109-1116, Phayathai Road, Pathumwan, Bangkok, 10330 Thailand; 2 Department of Epidemiology, Columbia University Mailman School of Public Health, New York, USA; 3 Thai Red Cross Anonymous Clinic, Thai Red Cross AIDS Research Centre, Bangkok, Thailand; 4 HIV-NAT, Thai Red Cross AIDS Research Centre, Bangkok, Thailand; 5 FHI 360, Bangkok, Thailand; 6 Center of Excellence in Transgender Health, Chulalongkorn University, Bangkok, Thailand

**Keywords:** Hepatitis B, Hepatitis C, HIV, Viral hepatitis vaccines, Sexual and gender minorities, Transgender persons

## Abstract

**Background:**

Viral hepatitis is highly prevalent among people with HIV (PWH) and can lead to chronic liver complications. Thailand started universal hepatitis B vaccination at birth in 1992 and achieved over 95% coverage in 1999. We explored the prevalence of hepatitis B and C viral infections and the associated factors among PWH from same-day antiretroviral therapy (SDART) service at the Thai Red Cross Anonymous Clinic, Bangkok, Thailand.

**Methods:**

We collected baseline characteristics from PWH enrolled in the SDART service between July 2017 and November 2019. Multivariable logistic regression was performed to determine factors associated with positive hepatitis B surface antigen (HBsAg) and hepatitis C antibody (anti-HCV).

**Results:**

A total of 4011 newly diagnosed PWH who had HBsAg or anti-HCV results at baseline: 2941 men who have sex with men (MSM; 73.3%), 851 heterosexuals (21.2%), 215 transgender women (TGW; 5.4%), and 4 transgender men (0.1%). Median age was 27 years. Overall seroprevalence of HBsAg and anti-HCV were 6.0 and 4.1%, respectively. Subgroup prevalence were 6.2 and 4.7% among MSM, 4.6 and 2.4% among heterosexuals, and 9.3 and 3.7% among TGW, respectively. Factors associated with HBsAg positivity were being MSM, TGW, born before 1992, CD4 count < 200 cells/mm^3^, and alanine aminotransferase ≥ 62.5 U/L. Factors associated with anti-HCV positivity were being MSM, age > 30 years, alanine aminotransferase ≥ 62.5 U/L, creatinine clearance < 60 ml/min, and syphilis infection.

**Conclusions:**

Around 5–10% of newly diagnosed PWH in Bangkok had hepatitis B viral infection after 25 years of universal vaccination. Anti-HCV positivity was found in 4–5% of PWH who were MSM and TGW. As World Health Organization and Thailand national guidelines already support routine screening of hepatitis B and C viral infections in PWH and populations at increased risk of HIV including MSM and TGW, healthcare providers should reinforce this strategy and provide linkage to appropriate prevention and treatment interventions. Catch-up hepatitis B vaccination should be made available under national health coverage.

## Background

Hepatitis B virus (HBV) and hepatitis C virus (HCV) infections are prevalent globally. In 2015, chronic HBV infection was estimated to be in 3.5% of the world population [1]. People with HIV (PWH) had a higher estimated prevalence rate of 7.4% [1]. In Thailand, a study estimated that 5.1% of the general population was infected with HBV in 2015 [2]. The proportion of infection was higher at 8.1% among men who have sex with men (MSM) and 8.1% among PWH [2]. A study conducted from 2006 to 2008 reported that 13.8% among MSM living with HIV were coinfected with HBV in Bangkok, Thailand [3]. Similarly, the global hepatitis C prevalence in 2015 was 1.0%, while 6.2% was the reported HCV prevalence among PWH. However, there is a heterogeneity of risk HCV infection among PWH populations: 82.4% in PWH who inject drugs, 6.4% in MSM living with HIV, and only 2.4% in PWH from the general population [1]. A national survey of the Thai general population in 2014 showed that hepatitis C antibody (anti-HCV) seroprevalence was 0.9% [4]. In contrast, a study focused on PWH found that anti-HCV seroprevalence was 7.7% [5], demonstrating that PWH shoulders a disproportionate burden of HCV infection when compared to the general population.

Furthermore, more than 70% of people infected with HCV and less than 6% of people who acquired HBV as an adult could turn into chronic carriers [1, 6]. Twenty per cent of chronic carriers could develop cirrhosis and hepatocellular carcinoma which are life threatening [1]. Having cofactors such as HIV infection and alcohol consumption can also accelerate the development of end-stage liver diseases [1, 6]. HBV and HCV are not only transmitted from mother to child but also sexually transmitted and share routes of transmission, including percutaneous and mucosal exposures [6, 7]. Having unprotected sex, multiple sexual partners, injection drug use, and a history of other sexually transmitted infections are important factors associated with HBV acquisition [6]. HCV is transmitted mainly through unsafe healthcare settings and injection drug use [1]. However, sexual transmission of HCV among PWH and MSM has been observed [6, 8–12]. MSM and transgender women (TGW) are prone to HCV infection due to the high prevalence of needle sharing, either for illicit drugs or cosmetic injections, and mucosal trauma associated with anal intercourse [9, 13–16].

Unlike HCV infection, HBV infection is a vaccine-preventable disease. Universal hepatitis B vaccination at birth was integrated into Thailand’s Expanded Program on Immunization (EPI) in 1992 and reached over 95% coverage in 1999 [17]. In 2014, a different proportion in HBV infection was seen among persons born before 1992 (4.5%) and persons born after 1992 (0.6%) [17]. Thailand national HIV treatment guidelines recommended baseline screening of newly diagnosed PWH with hepatitis B surface antigen (HBsAg) and anti-HCV, as HBV and HCV coinfections are commonly found and play a critical role in selecting antiretroviral therapy regimens [18].

The Thai Red Cross Anonymous Clinic (TRCAC) is the largest testing center for HIV and sexually transmitted infections in Bangkok, Thailand. Eligible clients who test positive for HIV are included in the same-day antiretroviral therapy (SDART) service [19]. Because this service includes clients of varied populations, namely heterosexuals, MSM, TGW, and transgender men (TGM), this study aims to explore prevalence and the associated risk factors of HBV and HCV seropositivity among PWH in the SDART service.

## Methods

This is a cross-sectional descriptive study collecting demographic and laboratory data of clients in the SDART service from 13 July 2017 to 30 November 2019. Included in this study were newly diagnosed PWH at TRCAC who tested for either HBsAg or anti-HCV at baseline. Demographic data included population category, age, birth year, educational level, residential location, and level of income. Persons born in or after 1992 were classified as “born after EPI,” and persons born before 1992 were “born before EPI.” This translates to the age of 25 years for individuals who tested in 2017, 26 in 2018, and 27 in 2019. Baseline laboratory tests included HBsAg, anti-HCV, CD4 count, alanine aminotransferase (ALT), creatinine clearance (CrCl), treponemal test, and nontreponemal test. A severe decrease in CD4 count was defined as a CD4 count of less than 200 cells/mm^3^ [20]. Regarding ALT, a cutoff of 62.5 U/L calculated from 1.25 times the upper normal limit of the local laboratory was used to indicate a mild increase [20]. Creatinine clearance was calculated with the Cockcroft-Gault equation and categorized as severely decreased if less than 60 ml/min [20]. Syphilis was diagnosed when the treponemal test (enzyme immunoassay, chemiluminescent microparticle immunoassay, rapid immunochromatographic assay, or treponema pallidum hemagglutination assay) and nontreponemal test (either rapid plasma reagin or venereal disease research laboratory) were both positive. Clients who did not initiate antiretroviral therapy at TRCAC might not have all baseline laboratory test results.

### Data analysis

Data were analyzed using Stata Version 15.0. Continuous parameters were presented as median with interquartile range (IQR). Categorical parameters were expressed in frequency and percentages. The prevalence of positive HBsAg and anti-HCV were calculated. Univariate and multivariable analyses were performed to determine factors associated with positive HBsAg and anti-HCV. Univariate analysis comparing HBsAg-positive PWH with HBsAg-negative using simple logistic regression model was tested. Factors with *p*-value less than 0.2 were selected into multivariable analysis. Multivariable analysis with a stepwise multiple logistic regression model including associated factors related to positive HBsAg or anti-HCV was conducted and reported with an adjusted odds ratio (aOR) and a 95% confidence interval (CI). A *p*-value of less than 0.05 was considered statistically significant.

## Results

The total number of PWH who had HBsAg or anti-HCV results at baseline was 4011 out of 6037: 4011 had HBsAg results available, while 3990 had anti-HCV results available. Most of them were MSM (73.3%), followed by heterosexuals (21.2%), TGW (5.4%), TGM (0.1%). Median age was 27 years (IQR 23–34). Sociodemographic and laboratory characteristics are shown in Table 1. Of the included clients, 46.0% were born after EPI, 25.9% had a CD4 count of less than 200 cells/mm^3^, and 19.3% had syphilis diagnosed at baseline.

**Table 1:** Distribution of HIV-Positive Individuals by Sociodemographic and Laboratory Characteristics

Characteristic	Total n (%) or value	HBsAg Positive n (%) or value	Anti-HCV Positive n (%) or value
**Sociodemographic data**			
Population	4011	242	164
MSM	2941 (73.3)	182 (75.2)	136 (82.9)
Heterosexual	851 (21.2)	39 (16.1)	20 (12.2)
TGW	215 (5.4)	20 (8.3)	8 (4.9)
TGM	4 (0.1)	1 (0.4)	0 (0.0)
Age (years), median (IQR)	27 (23–34)	31 (26–37)	29 (24–37)
Age (years)	4011	242	164
≤ 20	438 (10.9)	13 (5.4)	8 (4.9)
21–30	2112 (52.7)	107 (44.2)	81 (49.4)
31–40	1016 (25.3)	89 (36.8)	51 (31.1)
41–50	349 (8.7)	27 (11.2)	19 (11.6)
> 50	96 (2.4)	6 (2.5)	5 (3.0)
Birth year	4011	242	164
< 1992	2167 (54.0)	176 (72.7)	105 (64.0)
≥ 1992	1844 (46.0)	66 (27.3)	59 (36.0)
Birth year	4011	242	164
< 1999	3700 (92.2)	233 (96.3)	158 (96.3)
≥ 1999	311 (7.78)	9 (3.7)	6 (3.7)
Educational level	2320	138	112
High school or below	794 (34.2)	49 (35.5)	27 (24.1)
College or above	1526 (65.8)	89 (64.5)	85 (75.9)
Residential location	2019	116	99
Bangkok	1381 (68.4)	75 (64.7)	66 (66.7)
Outside Bangkok	638 (31.6)	41 (35.3)	22 (33.3)
Monthly income (baht)	1337	78	62
≤ 10,000	256 (19.1)	7 (9.0)	8 (12.9)
> 10,000	1081 (80.9)	71 (91.0)	54 (87.1)
**Laboratory data**			
HBsAg	4011	–	164
Positive	242 (6.0)	–	12 (7.3)
Negative	3769 (94.0)	–	152 (92.7)
Anti-HCV	3990	221	–
Positive	164 (4.1)	12 (5.4)	–
Negative	3826 (95.9)	209 (94.6)	–
CD4 count (cells/mm^3^)	4004	240	163
< 200	1038 (25.9)	86 (35.8)	37 (22.7)
≥ 200	2966 (74.1)	154 (64.2)	126 (77.3)
ALT (U/L)	3976	219	162
< 62.5	3623 (91.1)	177 (80.8)	98 (60.5)
≥ 62.5	353 (8.9)	42 (19.2)	64 (39.5)
CrCl (ml/min)	3923	213	160
< 60	29 (0.7)	0 (0.0)	5 (3.1)
≥ 60	3894 (99.3)	213 (100.0)	155 (96.9)
Syphilis	3972	225	163
Positive	767 (19.3)	54 (24.0)	54 (33.1)
Negative	3205 (80.7)	171 (76.0)	109 (66.9)

*HBsAg* hepatitis B surface antigen, *anti-HCV* hepatitis C antibody, *MSM* men who have sex with men, *TGW* transgender women, *TGM* transgender men, *ALT* aspartate aminotransferase, *CrCl* creatinine clearance

The overall prevalence of positive HBsAg was 6.0%. TGW had a prevalence of 9.3%, followed by MSM, 6.2%. People born after the EPI (1992 or later) had a prevalence of 3.6% as compared to 8.1% in people born before the EPI. Individuals born after the EPI reached more than 95% coverage (1999 or later) had a prevalence of 2.9% as compared to 6.3% in those born before 95% coverage. All nine clients who were born in 1999 or later and had a positive HBsAg were MSM.

The overall anti-HCV seroprevalence was 4.1%. MSM had the highest prevalence among all study population (4.7%). PWH aged over 30 years had a prevalence of 5.2%, while it was 3.5% among those younger than 30 years. Of 3990 who tested for both HBsAg and anti-HCV, 12 (0.3%) were positive for both. Overall and age-stratified prevalence of HBsAg and anti-HCV are shown in Table 2.

**Table 2:** Overall and Age-Stratified Prevalence of Hepatitis B Surface Antigen and Hepatitis C Antibody

Age (Years)	HBsAg		Anti-HCV	
	No. of HBsAg+ / No. of Tested	%	No. of Anti-HCV+ / No. of Tested	%
Overall	242/4011	6.0	164/3990	4.1
≤ 20	13/438	3.0	8/435	1.8
21–30	107/2112	5.1	81/2106	3.8
31–40	89/1016	8.8	51/1007	5.1
41–50	27/349	7.7	19/346	5.5
> 50	6/96	6.3	5/96	5.2

*HBsAg* hepatitis B surface antigen, *anti-HCV* hepatitis C antibody

Factors found significant at the 95% confidence level from the univariate analysis were carried over to the multivariable analysis as presented in Tables 3 and 4. Factors associated with positive HBsAg were being MSM (adjusted odds ratio [aOR] 1.64, 95% CI 1.13 to 2.40, *p* = 0.010), being TGW (aOR 2.87, 95% CI 1.60 to 5.17, *p* <  0.001), being born before 1992 (aOR 2.32, 95% CI 1.69 to 3.16, *p* < 0.001), CD4 count < 200 cells/mm^3^ (aOR 1.38, 95% CI 1.03 to 1.86, *p* = 0.031), and ALT ≥ 62.5 U/L (aOR 2.39, 95% CI 1.66 to 3.43, *p* <  0.001). Factors associated with positive anti-HCV were being MSM (aOR 2.11, 95% CI 1.26 to 3.55, *p* = 0.005), age > 30 years (aOR 1.54, 95% CI 1.10 to 2.17, *p* = 0.012), ALT ≥ 62.5 U/L (aOR 7.74, 95% CI 5.48 to 10.9, *p* <  0.001), CrCl < 60 ml/min (aOR 5.58, 95% CI 1.95 to 16.0, *p* = 0.001), and syphilis positive (aOR 1.95, 95% CI 1.36 to 2.78, *p* <  0.001).

**Table 3:** Hepatitis B Surface Antigen Prevalence in HIV-Positive Individuals and the Associated Factors Assessed by Univariate and Multivariable Analyses

Factor	No. of HBsAg+ / No. of Tested	%	Univariate		Multivariable^**†**^	
			Crude OR (95% CI)	***p***	Adjusted OR (95% CI)	***p***
Population	4011					
Heterosexual	39/851	4.6	Reference		Reference	
MSM	182/2941	6.2	1.37 (0.96 to 1.96)	0.079	1.64 (1.13 to 2.40)	0.010
TGW	20/215	9.3	2.14 (1.22 to 3.74)	0.008	2.87 (1.60 to 5.17)	< 0.001
TGM	1/4	25.0	6.94 (0.71 to 68.3)	0.097	5.77 (0.57 to 58.7)	0.138
Birth year	4011					
< 1992	176/2167	8.1	2.38 (1.78 to 3.18)	< 0.001	2.32 (1.69 to 3.16)	< 0.001
≥ 1992	66/1844	3.6	Reference		Reference	
Monthly income (baht)	1337					
≤ 10,000	7/256	2.7	Reference			
> 10,000	71/1081	6.6	2.50 (1.14 to 5.50)	0.023		
Residential location	2019					
Bangkok	75/1381	5.4	Reference			
Outside Bangkok	41/638	6.4	1.20 (0.81 to 1.77)	0.372		
Educational level	2320					
College or above	89/1526	5.8	Reference			
High school or below	49/794	6.2	1.06 (0.74 to 1.52)	0.743		
Anti-HCV	3990					
Negative	209/3826	5.5	Reference			
Positive	12/164	7.3	1.37 (0.75 to 2.50)	0.311		
CD4 count (cells/mm^3^)	4004					
< 200	86/1038	8.3	1.65 (1.25 to 2.17)	< 0.001	1.38 (1.03 to 1.86)	0.031
≥ 200	154/2966	5.2	Reference		Reference	
ALT (U/L)	3976					
< 62.5	177/3623	4.9	Reference		Reference	
≥ 62.5	42/353	11.9	2.63 (1.84 to 3.75)	< 0.001	2.39 (1.66 to 3.43)	< 0.001
CrCl (ml/min)	3923					
< 60	0/29	0.0	–			
≥ 60	213/3894	5.5	–	–		
Syphilis	3972					
Negative	171/3205	5.3	Reference			
Positive	54/767	7.0	1.34 (0.98 to 1.84)	0.067		

*HBsAg* hepatitis B surface antigen, *CI* confidence interval, *anti-HCV* hepatitis C antibody, *ALT* alanine aminotransferase, *CrCl* creatinine clearance

^†^*n* = 3974

**Table 4:** Hepatitis C Antibody Prevalence in HIV-Positive Individuals and the Associated Factors Assessed by Univariate and Multivariable Analyses

Factor	No. of Anti-HCV+ / No. of Tested	%	Univariate		Multivariable^**†**^	
			Crude OR (95% CI)	***p***	Adjusted OR (95% CI)	***p***
Population	3990					
Heterosexual	20/849	2.4	Reference		Reference	
MSM	136/2923	4.7	2.02 (1.26 to 3.25)	0.004	2.11 (1.26 to 3.55)	0.005
TGW	8/214	3.7	1.61 (0.70 to 3.71)	0.263	1.78 (0.74 to 4.30)	0.199
TGM	0/4	0.0	–	–	–	–
Age	3990					
≤ 30	89/2541	3.5	Reference		Reference	
> 30	75/1449	5.2	1.51 (1.10 to 2.06)	0.011	1.54 (1.10 to 2.17)	0.012
Monthly income (baht)	1330					
≤ 10,000	8/256	3.1	Reference			
> 10,000	54/1074	5.0	1.64 (0.77 to 3.49)	0.199		
Residential location	1999					
Bangkok	66/1367	4.8	Reference			
Outside Bangkok	33/632	5.2	1.09 (0.71 to 1.67)	0.706		
Educational level	2301					
College or above	85/1514	5.6	Reference	0.022		
High school or below	27/787	3.4	0.60 (0.38 to 0.93)			
CD4 count (cells/mm^3^)	3985					
< 200	37/1027	3.6	Reference			
≥ 200	126/2958	4.3	1.19 (0.82 to 1.73)	0.360		
ALT (U/L)	3975					
< 62.5	98/3623	2.7	Reference		Reference	
≥ 62.5	64/352	18.2	7.99 (5.71 to 11.2)	< 0.001	7.74 (5.48 to 10.9)	< 0.001
CrCl (ml/min)	3923					
< 60	5/29	17.2	5.03 (1.89 to 13.3)	0.001	5.58 (1.95 to 16.0)	0.001
≥ 60	155/3894	4.0	Reference		Reference	
Syphilis	3965					
Negative	109/3203	3.4	Reference		Reference	
Positive	54/762	7.1	2.16 (1.55 to 3.03)	< 0.001	1.95 (1.36 to 2.78)	< 0.001

*Anti-HCV* hepatitis C antibody, *CI* confidence interval, *ALT* alanine aminotransferase, *CrCl* creatinine clearance

^†^*n* = 3901

## Discussion

Among PWH who were newly diagnosed at the Thai Red Cross Anonymous Clinic, we found 6% prevalence of HBV and 4% prevalence of anti-HCV positivity. Being MSM, TGW, and born before the inclusion of universal hepatitis B vaccination in Thailand’s EPI were statistically associated with HBV infection. Moreover, being MSM and having syphilis increased the chance of being anti-HCV positive.

More than 70% of our clients were MSM, and 5% were TGW. These findings are consistent with the proportions of new HIV infections in Thailand as projected by the AIDS Epidemic Model [21]. We found the HBV prevalence to be highest among TGW (9%), followed by MSM (6%). The anti-HCV seroprevalence was highest among MSM (5%), followed by TGW (4%). These data support the findings from previous studies which demonstrated higher prevalence rates of HBV and HCV among PWH who were MSM and TGW than those who were of heterosexual populations [1, 3, 15, 22–24]. The HBV prevalence shown in our clients is quite concerning, as 27% of HBV-infected individuals were born after EPI, indicating that the HBV epidemic is still ongoing. However, among clients infected with HBV, only 4% were born in 1999 or later, which was the year that EPI reached more than 95% coverage. Lower prevalence of HBV in younger generations could be due to both the success of EPI and shorter duration at risk. The overall prevalence of HBV and HCV found in our study were slightly lower than that of the global and Thai reports among PWH [1, 2, 5], possibly because our clients were relatively young with over 60% aged 30 years or less . Apart from viral hepatitis, almost 20% of our clients had syphilis at baseline, illustrating higher burden than what was previously reported in general MSM population by World Health Organization (WHO; 6%) in 2018 [25].

MSM and TGW had 1.6 and 2.9 times the odds of HBV infection compared with heterosexual populations, respectively, which could be explained by sexual practices and routes which are more vulnerable to mechanical trauma [26]. Consistent with the national survey [17], we saw a contrast of HBV prevalence between people who were born before and after EPI (8% vs. 4%). PWH who were born before EPI had 2.3 times higher odds of having HBV infection. Another possible explanation of the association to birth year could be that younger people had less time for exposure to the infection than older people. Nonetheless, the findings from this study shows that HBV infection was not completely eliminated by the EPI. Some newborns might be born to an HBV-infected mother, not complete the full course of the immunization, not respond to HBV vaccination, or be unvaccinated. Regarding PWH who do not have immunity to HBV, it is advised to immunize all PWH regardless of CD4 level [18], even though lower CD4 count is one of the factors affecting the effectiveness of the HBV immunization [27–29].

There are many reports of the HCV epidemic among MSM living with HIV in major cities of the world in Europe, Asia-Pacific, and Thailand [8–10, 30–36], and many findings demonstrated an upward trend of HCV infection [9, 30–36]. For example, a study in Thailand found that HCV incidence increased from 0.7–1.1 per 100 person-years in 2014–2016 to 4.5 per 100 person-years in 2018 [30]. Our findings also indicated similar discovery, as we found that being MSM and having syphilis doubled the risk of having positive anti-HCV in our study. HCV infection is widely known to be associated with recreational drug use [9, 10, 30, 31, 35, 37–40], as some MSM utilize recreational drugs to enhance their sexual pleasure, the phenomenon known as *chemsex* [41, 42]. Studies on people who did not use injection drugs found that HCV infection was still associated with recreational drug use and syphilis [33, 35, 43], suggesting that transmission of HCV is associated with sexual intercourse. It has been estimated that the prevalence of drug injection among newly diagnosed Thai PWH in 2020 is 12% [44]. Unfortunately, we did not collect data on substance use and chemsex in our study and could not assess these potential associations. However, as MSM and syphilis were key factors associated with HCV infection among our clients, we hypothesized that HCV acquisition in our HIV-positive MSM clients was likely linked to sexual transmission and possibly in the chemsex context.

There were a few limitations in our study. This study was conducted at one site and two-thirds of our PWH clients lived in Bangkok, suggesting that they may be more educated and had higher income when compared to PWH from other regions in Thailand. Thus, our study sample might not represent the overall population of PWH in Thailand. Additionally, we could not retrieve hepatitis B vaccination history from original medical records, and therefore needed to use PWH clients’ birth year as a surrogate. As previously mentioned, we did not record substance use and chemsex data and thus could not explore their possible associations with HBV and HCV infections in our study. Lastly, we did not perform HCV RNA in anti-HCV-positive clients; therefore, the observed anti-HCV seroprevalence may be higher than the actual infection rate.

WHO aimed to eliminate hepatitis B and C infections by 2030 [45]. To achieve this, a country must target the areas determined to have beneficial impacts as described in Table 5 [45, 46]. In Thailand, HBV vaccination has reached 95% coverage since 1999 [17]. However, Thailand’s diagnosis and treatment coverage of HBV and HCV are far from the target [46]. WHO and Thailand already recommend HBV and HCV infection screening in PWH and those who are at risk of HIV including MSM and transgender people [18, 47, 48]. From the results of this study, continuation of HBV and HCV screening for everyone living with HIV regardless of age would uncover a high proportion of undiagnosed infections and be a chance to bring them to treatment. Providing HIV preexposure prophylaxis to HIV-negative persons at risk of HIV acquisition and following the current WHO and Thailand guidance to test for HIV, HBV, and HCV infections at commencement of the services can be an opportunity to screen, vaccinate (HBV), and if needed, treat these infections to prevent further transmission [49, 50]. In spite of that, catch-up hepatitis B immunization needs to be paid out of pocket for in Thailand. Regarding HCV, some global HCV elimination efforts have now included pangenotypic direct-acting antiretroviral (DAA) therapy for most HCV-infected individuals [51, 52]. In Thailand, costs of HCV genotyping and treatment have been high, but the voluntary licensing of DAA medications is becoming implemented in Thailand [53]. However, the current restriction to only start DAA in individuals with fibrosis METAVIR Stage F2 means that individuals with HCV can continue to transmit the virus to others. Future studies will need to explore strategies to timely and efficiently test and treat HCV, especially among PWH, to contribute to the elimination of HCV.

**Table 5:** Global Baseline and Thailand’s Progress Toward the Elimination of Hepatitis B and C by 2030

Target Area	Global Baseline 2015	Thailand Estimates 2019	WHO Target 2030
HBV birth dose	38%	99%	90%
HBV 3+ doses	82%	97%	90%
Blood safety	89%	100%	100%
Injection safety	5%	100%	90%
Syringes per PWID^†^	20	24	300
HBV diagnosed	< 5%	7%	90%
HBV treated	< 1%	1%	80%
HCV diagnosed	< 5%	36%	90%
HCV treated	< 1%	11%	80%

*WHO* World Health Organization, *HBV* hepatitis B virus, *PWID* person who injects drugs, *HCV* hepatitis C virus

^†^Number of sterile needles and syringes provided per person who injects drugs per year

## Conclusions

Thailand’s EPI has successfully reduced HBV infection. However, the infection rate among newly diagnosed PWH remained at around 5–10%. Hepatitis C infection was found in 4–5% of PWH who were MSM and TGW. Healthcare providers should reinforce HBV and HCV screening in PWH, MSM, and TGW and provide linkage to appropriate prevention and treatment interventions. Catch-up hepatitis B vaccination should be made available under national health coverage.

## Data Availability

The datasets used and/or analyzed during the current study are available from the corresponding author on reasonable request.

## Abbreviations

aOR: Adjusted odds ratio
CI: Confidence interval
CrCl: Creatinine clearance
ALT: Alanine aminotransferase
Anti-HCV: Hepatitis C antibody
DAA: Direct-acting antiretroviral
EPI: Expanded Program on Immunization
HBsAg: Hepatitis B antigen
HBV: Hepatitis B virus
HCV: Hepatitis C virus
IQR: Interquartile range
MSM: Men who have sex with men
PWID: Person who injects drug
PWH: People with HIV
SDART: Same-day antiretroviral therapy
TGW: Transgender women
TRCAC: Thai Red Cross Anonymous Clinic
WHO: World Health Organization
